# Evidence-based selection of environmental factors and datasets for measuring multiple environmental deprivation in epidemiological research

**DOI:** 10.1186/1476-069X-8-S1-S18

**Published:** 2009-12-21

**Authors:** Elizabeth A Richardson, Richard J Mitchell, Niamh K Shortt, Jamie Pearce, Terence P Dawson

**Affiliations:** 1 School of GeoSciences, The University of Edinburgh, Drummond Street, Edinburgh EH8 9XP, UK; 2 Section of Public Health and Health Policy, University of Glasgow, 1 Lilybank Gardens, Glasgow G12 8RZ, UK; 3 School of Geography, University of Southampton, Highfield, Southampton SO17 1BJ, UK

## Abstract

This Environment and Human Health project aims to develop a health-based summary measure of multiple physical environmental deprivation for the UK, akin to the measures of multiple socioeconomic deprivation that are widely used in epidemiology. Here we describe the first stage of the project, in which we aimed to identify health-relevant dimensions of physical environmental deprivation and acquire suitable environmental datasets to represent population exposure to these dimensions at the small-area level. We present the results of this process: an evidence-based list of environmental dimensions with population health relevance for the UK, and the spatial datasets we obtained and processed to represent these dimensions. This stage laid the foundations for the rest of the project, which will be reported elsewhere.

## Introduction

The physical environment is multifactorial. Environmental factors with health relevance range from pathogenic (i.e., with potential to damage health), to salutogenic (i.e., with potential to enhance or maintain health). Constrained by data limitations and availability, studies of the health effects of the environment often focus on a subset of the environment in isolation [e.g., air pollution: [1]]. However, populations are not exposed to single environmental factors in isolation: they simultaneously experience multiple exposures. Evans and Kantrowitz [p.304; [2]] suggest that "... multiple exposures to a plethora of suboptimal environmental conditions" may help explain socioeconomic inequalities in health. Different environmental exposures may have additive, synergistic or antagonistic effects on health when experienced in combination [3], hence identifying areas experiencing multiple environmental deprivation may assist in clarifying environment and health relationships and our understanding of health inequalities.

Composite indicators, or indices, are used in other disciplines to present information from multiple variables in an understandable and usable form [4]. A UK example is the Carstairs score [5], which summarises four elements of multiple socioeconomic deprivation: material possessions, employment, living conditions and social class. Socioeconomic deprivation indices such as the Carstairs score are widely used in epidemiology, and have greatly facilitated research into the relationships between socioeconomic deprivation and health [6].

There is a growing need for measures that summarise environmental influences on health in a meaningful way, in order to inform policy-making and interventions [4]. Environmental summary measures have been trialled elsewhere (e.g., [7,8]), but none have sought to summarise multiple environmental deprivation in a specifically health-relevant way. A carefully constructed measure summarising health-relevant aspects of the physical environment could help improve our understanding of the importance of environmental determinants of health.

Our larger project aimed to quantify the overall health-related environmental burden faced by a population, by developing a health-based summary measure of multiple physical environmental deprivation for the UK and determining its utility. Here we describe the first stage of the project, in which we aimed to identify health-relevant dimensions of physical environmental deprivation and acquire suitable environmental datasets. Due to word constraints we summarise key decisions made and our justification for them. We also present the results of this process: an evidence-based list of environmental dimensions with population health relevance for the UK, and the spatial datasets we obtained and processed to represent these dimensions. Subsequent methodological steps, analysis and interpretation that builds on the work described here will be presented elsewhere.

## Methods

### Stage 1: Identifying health-relevant dimensions of environmental deprivation

A summary measure of health-related physical environments should include only factors with a clear association with health [4], and to which substantial numbers of people are exposed. We therefore began by identifying distinct dimensions of physical environmental deprivation that were public health-relevant and quantifiable for the UK. A crucial first step was defining the 'physical environment': we decided to include external physical, chemical and biological factors and exclude social and cultural factors.

We documented the reasons for decisions made during the process in order to maximise transparency and promote replicability. We initially conducted a scoping review to guide selection of health-relevant environmental factors. To avoid overlooking less commonly-researched factors we consulted a diverse range of sources. A long list of potentially relevant environmental factors was produced using a wide range of international academic and grey literature and by browsing titles returned from general 'environment + health' searches of publication databases (PubMed, WebOfKnowledge and GeoBase). For public health relevance we required that at least 10% of the UK population was exposed to each environmental factor; the factors for which this threshold would not be met were excluded.

We then systematically searched publication databases for empirical studies that had explored the health impacts of one or more environmental factors on the long list. Search terms were derived from the long list. The WebOfKnowledge database allowed us to order search results by citation scores, ensuring that key references were not overlooked. Additionally, reference lists of papers were manually browsed to locate further studies. Non-English language studies and those pre-1980 were excluded. Searching was halted for each environmental factor when a saturation point was reached, i.e., when no novel results were being returned.

The assembled evidence was appraised by the project team during group discussions, based on prevalence of the health outcome(s), rigour of the study design, and the strength of association established. Spatial datasets were sought to enable assessment of population exposure to factors when exceedance of the 10% threshold was not apparent from the literature. The end-product of this stage was a wish-list of health-relevant environmental factors we would want to include in our summary measure.

### Stage 2: Dataset acquisition and processing

To maximise future utility and reproducibility of the measure the datasets used would ideally be readily available, routinely updated, representative of the environmental factors of concern and of an acceptable and comparable quality [7,9]. For each environmental factor on our wish-list we therefore sought data that were spatially contiguous, comprehensive across the UK and centred around 2001, to correspond with the decennial census which would be our source of denominator data for subsequent testing of the summary measure's utility. The environmental data needed to be fit for the purpose of reliably representing long-term exposure to each factor.

We selected UK 2001 Census Area Statistics (CAS) wards as our geographical unit of analysis. There were 10,654 CAS wards in the UK at the 2001 census, with an average population of approximately 5,500. Using the geographical information system software ArcMap (ESRI Inc., Redlands, CA) we rendered each environmental dataset to the 2001 CAS wards.

## Results

We long-listed 13 environmental factors and appraised the evidence for i) their association with health outcomes using the international literature and ii) their relevance to population health in the UK context. Consequently, seven factors were included in our wish-list for our summary measure of environmental deprivation (Table 1). Table 1 briefly outlines some key epidemiological evidence for the health associations of each wish-listed factor (from meta-analyses where available), although the full evidence review for each factor was more comprehensive than can be reported here. Six environmental factors were excluded (Table 2) as a result of this evidence appraisal process.

**Table 1:** Summary justification for the environmental factors selected for our wish-list, including examples of typical effect sizes.

Environmental factor	Examples of typical risks reported (+ 95% CI)
*Outdoor ambient air pollutants* Elevated risks of respiratory disease (RD), cardiovascular disease (CVD) and total mortality consistently associated with air pollutants, at concentrations frequently experienced in urban settings [11-16]. Evidence of health effects strongest for particulate matter (PM_10_) and ozone (O_3_), but also substantial for carbon monoxide (CO), sulphur dioxide (SO_2_) and nitrogen dioxide (NO_2_).	Meta-analysis all-cause mortality RR for 10 μg.m^-3 ^increase in pollutant [17]: PM_10 _= 1.006 (1.004 to 1.008) (33 studies) O_3 _= 1.003 (1.001 to 1.004) (15 studies) Meta-analysis % excess mortality for stated increase [11]: CO = 1.7 (1.2 to 2.2) for 1.1 ppm increase (22 effect estimates) SO_2 _= 0.9 (0.7 to 1.2) for 9.4 ppb increase (46 effect estimates) NO_2 _= 2.8 (2.1 to 3.5) for 24.0 ppb increase (32 effect estimates)

*Climate* Increased risks of CVD, RD and total mortality with both elevated and reduced temperatures found in many studies. [18-20]. Small but persistent elevations in risk are seen with each incremental deviation away from the UK's comfort temperature of 20°C [20], hence the entire population are exposed.	RD mortality: Cold: Six European country study, 2.46% (1.81 to 3.12) increase per 1°C drop below 18°C [21] Heat: The Netherlands, 10.4% (0.0 to 20.8) increase per 1°C increase above 16.5°C [22] London, 5.44% (1.92 to 9.09) increase per 1°C increase above 23°C [23]

*Solar ultraviolet (UV) radiation* UV radiation is the main risk factor for skin cancer [24,25], but a consistent protective effect of UV (via vitamin D production) has been found against a number of more prevalent cancers [25-27]. All studies on prostate, breast and ovarian cancer that were systematically reviewed by van der Rhee et al. [26] showed a significant inverse relationship between sunlight and mortality/incidence. Most of the UK population experience some vitamin D deficiency in winter because of inadequate exposure to solar UV [28].	Skin cancer: summary OR (29 studies) for maximally exposed subjects (non-occupational exposure) = 1.71 (1.54 to 1.90) [24] Mortality OR for high vs. low sunlight exposure [29]: Prostate cancer 0.90 (0.87 to 0.93) (97,873 cases) Breast cancer 0.74 (0.72 to 0.76) (130,261 cases) Ovarian cancer 0.84 (0.81 to 0.88) (39,002 cases) Colon cancer 0.73 (0.71 to 0.74) (153,511 cases)

*Industrial facilities* There is evidence that residence within 4 km of waste management sites or within 1.6 km of metal production/processing plants increases some cancer risks [e.g., [30-32]]. Evidence was inconsistent for refineries and combustion installations, and weak or non-existent for other facilities, therefore we included only waste management and metal production/processing sites. Analysis using a geographical information system (GIS) revealed that 21% of the UK population resided within the relevant effect buffers reported for these sites.	Within approx. 1.6 km of metal works: HR all-cause mortality for boys resident ≥ 10 yr = 1.52 (significantly elevated) [33] RR lung cancer mortality (vs. > 6 km) = 5.0 (1.4 to 17.8) [32] Cancer incidence RR within approx. 4 km of waste site (vs. low exposure): Non-Hodgkin's lymphoma = 2.3 (1.4 to 3.8) [34] Stomach cancer = 1.27 (1.04 to 1.55) [30]

*Green space* There is evidence that more natural environments have a beneficial effect on people's self-perceived health, blood pressure, levels of overweight and obesity and total mortality risks [35-42]. Population exposure to green space varies markedly across the UK and there is no indication of a minimum threshold for health.	IRR for high vs. low green space exposure [39]: All-cause mortality 0.94 (0.93 to 0.96) Circulatory disease mortality 0.96 (0.93 to 0.99)

*Drinking water quality* Disinfection By-Products (DBPs) have been consistently associated with a small elevated risk of bladder cancer, the fourth most common cancer in the UK [43,44]. Most of the UK population is exposed to disinfected drinking water.	Meta-analysis OR for cancer incidence (vs. low exposure): Bladder cancer = 1.1 (1.0 to 1.2) for intermediate exposure and 1.4 (1.2 to 1.7) for long-term exposure (>40 years) (8 studies) [44] All cancer = 1.15 (1.09 to 1.20) for high exposure (12 studies) [43]

*Noise* Strong associations found with ischaemic heart disease and hypertension, both of which increase mortality risk [45].	Meta-analysis RR for 5 dB(A) increase in noise [45]: Hypertension with occupational noise = 1.14 (1.01 to 1.29) Hypertension with air traffic noise = 1.26 (1.14 to 1.39) Ischaemic heart disease with road traffic noise = 1.09 (1.05 to 1.13)

CI = confidence interval, HR = hazards ratio, IRR = incidence rate ratio, OR = odds ratio, RR = relative risk.

**Table 2:** Summary justification for the environmental factors considered but excluded from our wish-list.

**Environmental factor**

*Extremely Low Frequency (ELF) radiation (power lines)* Studies find elevated rates of childhood leukaemia, the most common childhood cancer, with effects seen within 600 m of power lines [43,44,46]. Analysis of the National Grid for England and Wales (using GIS) revealed < 8% population exposure within 600 m.

*Radio Frequency (RF) radiation (radio and TV transmitters)* Inconsistent evidence for cancer effect [47]. Effects found within 10 km of the most powerful transmitters [48], of which there are approximately 20 in the UK. Population exposure < 10%.

*Radon* Strong evidence found for association with lung cancer [49,50], and an estimated 9% of lung cancer cases in Europe are attributable to radon exposure [49]. However, datasets for different regions of the UK have been prepared using different methodologies and at different resolutions, and population exposure to radon levels above the Action Level of 200 Bq/m^3 ^in England and Wales (highest resolution data) is < 4% (our own GIS analysis).

*Individual industrial pollutants* Although there is evidence for the health effects of acute (accidental or occupational) exposure to specific hazardous chemicals [51], there is little or no evidence for their health effects at environmental levels.

*Nuclear facilities* Evidence for an association with health is inconsistent and not strong [52,53]. A small proportion of the population lives in the proximity of the UK's 27 nuclear installations.

*Contaminated land* The primary route of exposure for many soil contaminants is consumption of soil or contaminated vegetables [54], so << 10% population exposure. Mapping contaminated land for the UK is incomplete [55], and modelling human exposure to contaminants in soil is a highly complex process [56].

**Figure 1:**
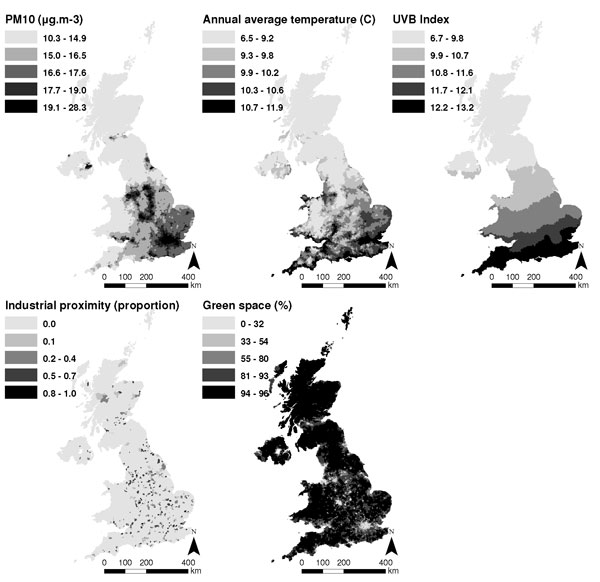
Ward-level measures of the environmental factors, separated into equal quintiles (by number of wards).

We attempted to obtain UK-wide datasets representative of the environment in 2001 (the date for which reliable population data are available) for the seven wish-listed factors. However, data reliability and availability issues meant that we were unable to obtain suitable information pertaining to drinking water quality and noise. For the five remaining environmental factors, the UK-wide datasets obtained and the ward-level measures derived from these data are detailed in Table 3. Mapping each measure in a geographical information system (GIS) (Figure 1) confirmed that the datasets represented expected geographical trends (e.g., higher pollution in urban areas, and higher average temperatures towards the south and east).

**Table 3:** Details of the datasets acquired and ward-level measures derived for the key environmental factors.

Key factor	Specific aspect	Data source	Ward-level measure derived
Air pollution	Particulate matter (PM_10_) Ozone (O_3_) Nitrogen dioxide (NO_2_) Sulphur dioxide (SO_2_) Carbon monoxide (CO)	AEA Technology (1 km grids, annual average concentrations, modelled from National Atmospheric Emissions Inventory (NAEI) data, 1999-2006)	Population-weighted average of each pollutant (averaged 1999 to 2003 for all except CO: 2001 to 2006)

Climate	Average temperature Cooling degree-days^1^ Heating degree-days^2^ Winter coldwave duration^3^ Summer heatwave duration^4^	Meteorological Office UK Climate Impact Programme data (5 km grids, 1996-2003)	Population-weighted average of each climate variable (averaged 1999 to 2003, except for coldwaves and heatwaves: 1996-2000)

UV radiation	-	UVB Index [57] calculated using Meteorological Office monthly cloud cover data (1 km grid, 1991-2000) and latitude	Population-weighted average UVBI (1991 - 2000)

Industrial facilities	Waste management sites Metal production/processing sites	European Pollutant Emission Register (EPER) (grid references, 2001-2002)	Proportion of population living within 4 km of waste site or 1.6 km of metal site (2001 - 2002)

Green space	-	Generalised Land Use Database (GLUD, England only, 2001) and Coordination of Information on the Environment (CORINE) Land Cover Data (UK, 2000)	Modelled GLUD % green space using CORINE variables and population density (2001) (R^2 ^= 0.95), then used model to predict % green space for whole UK

^1 ^no. of degree-days above 22°c

^2 ^no. of degree-days below 15.5°c in winter months

^3 ^no. of days with daily minimum >3°C below 1961-90 daily normal for ≥5 consecutive days (Nov-Apr)

^4 ^no. of days with daily maximum >3°C above 1961-90 daily normal for ≥5 consecutive days (May-Oct)

## Discussion

We aimed to identify health-relevant dimensions of physical environmental deprivation and acquire suitable environmental datasets. Guided by accepted principles for the design of composite indicators [4], we have documented the rationale behind our decisions throughout, to ensure transparency and repeatability.

The summary measures we will create and test during subsequent phases of the project will only be as good as the datasets we have used, hence we sought the most reliable data available. We were only able to obtain reliable and contiguous data for five of the seven environmental factors on our wish-list, but the list remains as a useful by-product of the process. The excluded wish-list factors (drinking water quality and noise) could be included in future attempts at summarising multiple environmental deprivation should suitable datasets become available. Furthermore, based on evidence of effect size we judged that the health impacts of the two excluded factors were unlikely to be as substantial as, say, air pollution, and hence anticipated that a useful composite indicator could be constructed using the remaining five factors.

Additionally, the acquired datasets had limitations. To ensure the future utility and replicability of the work we prioritised datasets that were readily available and likely to be routinely updated in the future. The pollutant and meteorological datasets selected for meeting these criteria were relatively coarse grids (1 and 5 km), therefore they may not adequately represent individual-level exposure in each ward. Although finer resolution data may have helped alleviate these exposure misclassification issues it is less likely that any such data, if available, would be readily available for the whole UK and/or routinely updated.

The preparation of separate datasets by the four countries of the UK, sometimes to different standards, also proved problematic. In particular the detailed land use mapping product (Generalised Land Use Database, GLUD) was only available for England, whereas the coarser resolution dataset (Coordination of Information on the Environment, CORINE) was available for the whole UK. For industrial facility locations the readily available EPER database (EU-wide) usefully combined all UK data. Whilst this circumvented the need for us to acquire different datasets from separate organisations, and would aid future reproducibility of the work, EPER data from the first reporting year (2001) are known not to be fully comprehensive for all facilities in all countries [10]. Future work may therefore have more comprehensive data to incorporate.

Where necessary our environmental datasets were averaged to give an indication of long-term exposure in each ward. However, short-term variations in some environmental factors have health-relevance (e.g., air pollutant peaks, extreme low temperature events). Implicit in our approach, therefore, is the assumption that the severity of the short-term extremes correlates well with longer-term averages.

## Conclusion

We have demonstrated that it is possible to identify health-relevant dimensions of the physical environment and acquire suitable datasets to represent variation in these dimensions across the UK. In doing so we have laid the foundations for the rest of the project: constructing a summary measure of multiple environmental deprivation for the UK and determining its utility in researching spatial inequalities in health. The process and its limitations have been transparently described, to aid further work and the informed use of its outputs.

## List of abbreviations used

AEA: Atomic Energy Authority; CA: California State; CAS: Census Area Statistics; CI: Confidence interval; CORINE: Coordination of Information on the Environment; CVD: Cardiovascular disease; DBP: Disinfection By-Products; ELF: Extremely Low Frequency radiation; EPER: European Pollutant Emission Register; ESRI: Environmental Systems Research Institute; EU: European Union; GIS: Geographical Information System; GLUD: Generalised Land Use Database; HR: Hazards ratio; NAEI: National Atmospheric Emissions Inventory; OR: Odds ratio; RD: Respiratory disease; RF: Radio Frequency radiation; RR: Relative risk; UK: United Kingdom; UV: Ultraviolet.

## Competing interests

The authors declare that they have no competing interests.

## Authors' contributions

ER prepared the reviews and drafted the manuscript. ER acquired and processed datasets, assisted by TD. RM conceived of the study. All authors helped collate literature. All authors participated in design and coordination of the study, and helped review the empirical evidence. All authors read and approved the final manuscript.

## Note

The peer review to this article can be found in Additional file 1.

## Supplementary Material

Additional file 1 Peer review. Click here for file
