# Supplementary material for: Evidence-based selection of environmental factors and datasets for measuring multiple environmental deprivation in epidemiological research
Source: Environ Health. 2009 Dec 21;8(Suppl 1):S18. doi: 10.1186/1476-069X-8-S1-S18 (PMC2796496; doi:10.1186/1476-069X-8-S1-S18)
Supplement: Additional file 1 — Peer review. [file 1476-069X-8-S1-S18-S1.doc]

**RESPONSE TO REVIEWERS:**

| Article ref no. | A1471-2105X SZ-S18 |
| --- | --- |
| Title | Evidence-based selection of environmental factors and datasets for measuring multiple environmental deprivation in epidemiological research |
| Author(s) | Elizabeth A. Richardson, Richard J. Mitchell, Niamh K. Shortt, Jamie Pearce, Terence P. Dawson |

- We thank the reviewers for their considered comments. Whilst mindful of the word limit, we have addressed the issues raised. We detail our response to the reviewers’ comments below.

**Ref#1**

**General comments:**

This is an interesting paper laying out the rationale for what to include in a geographically based index of local environmental exposures, to be linked to population census data. As a description of what the team has decided to include, this paper will have utility depending on the success of the index itself. Without details of the index, it is difficult to anticipate what aspects of this background documentation might be deficient. It may therefore be more helpful to readers to publish this and the index paper together.

We agree that a longer, more detailed paper will provide the broader context of our research but we were limited by the word constraints. We therefore restricted our aim for this paper: to identify health-relevant dimensions of physical environmental deprivation and acquire suitable datasets. Further methods and results papers from the project are either in review or close to submission, hence will be available in due course. Nonetheless we feel that a paper that outlines the decision making process involved in laying the evidence-based foundations for the development of environmental summary measures is an important addition to the literature.

**Discretionary revisions:**

A core area of controversy is likely to be whether the evidence for specific exposures affecting health is actually solid enough. A second issue is likely to be whether the correct weight is applied to individual factors when combining them into an index. The paper could therefore be strengthened by a more explicit tabulation of the nature of the evidence for the included factors and the magnitudes of effects. At the moment we are offered a few references and a qualitative statement of the evidence – extra columns summarising the nature of the evidence and the size of the effects for specific population groups and outcomes, plus notes on controversies over the evidence would be more helpful. The authors may feel that this is too big a canvas, but for an index to be meaningful each element needs to be evidence based and properly weighted.

- We have followed the advice by adding some typical effect sizes from the literature we reviewed (Table 1). The additional column provides a clearer rationale for our decisions. We emphasise that this still only provides a summary of the more comprehensive review we undertook (p 6).
- The second issue raised (applying correct weights) is addressed in a subsequent paper that details our construction of summary measures of multiple environmental deprivation. The current paper did not aim to address how to combine the environmental datasets, due to space constraints, hence we feel that discussion of weightings here would be premature.

**Major compulsory revisions:**

None

**Minor essential revisions:**

A more robust discussion of the limitations of this work should be presented. Relating geography of residence to local risks or amenities (green space) may be a poor proxy for actual individual or population exposures. Even personal exposure to radon may vary greatly in the same geographical area depending on the use of simple venting of buildings.

The discussion of evidence suggests that each risk is specific for rather isolated health impacts – could you discuss how combining such disparate exposure – disease relationships is conceptually meaningful.

- We acknowledge that these are limitations of the application of our summary measures in epidemiological research. However, they are not limitations of the work presented in this specific paper: i.e., the identification of health-relevant environmental factors and the acquisition and processing of suitable datasets. Limitations of subsequent stages in the work will be discussed in the relevant papers.

**Ref#2**

**General comments:**

This paper is a background for the development of a geographical environmental exposures index to link to population data that is to be reported in another publication. While this is a worthwhile exercise, the absence of any results renders the paper difficult to assess. The authors are probably faced with a dilemma they wish to publish their findings in full in another journal.

**Major compulsory revisions:**

Needs to include at least some example data from the findings.

- We have now added a results section to present i) the evidence for the health-relevant dimensions we identified, and ii) a summary (including maps) of the datasets we acquired (p 6).
